# Reduced mortality from KPC-*K.pneumoniae* bloodstream infection in high-risk patients with hematological malignancies colonized by KPC-*K.pneumoniae*

**DOI:** 10.1186/s12879-021-06747-8

**Published:** 2021-10-19

**Authors:** Alessandra Micozzi, Giuseppe Gentile, Stefania Santilli, Clara Minotti, Saveria Capria, Maria Luisa Moleti, Walter Barberi, Claudio Cartoni, Silvia Maria Trisolini, Anna Maria Testi, Anna Paola Iori, Giampaolo Bucaneve, Robin Foà

**Affiliations:** 1 Department of Translational and Precision Medicine, Sapienza University of Rome, Via Benevento 6, 00161 Rome, Italy; 2 Department of Diagnostics, Azienda Policlinico Umberto I, Rome, Italy; 3 Department of Hematology, Oncology and Dermatology, Azienda Policlinico Umberto I, Rome, Italy; 4 Ospedale S. Maria Della Misericordia, Perugia, Italy

**Keywords:** KPC-*K.pneumoniae*, Hematological malignanciy, Carriers, Bacteremia, Mortality, Initial active treatment

## Abstract

**Background:**

KPC-*K.pneumoniae* bloodstream infection (KPC-KpBSI) mortality rate in patients with hematological malignancies is reported about 60%. The initial treatment active against KPC-*K.pneumoniae* is crucial for survival and KPC-*K.pneumoniae* rectal colonization usually precedes KPC-KpBSI. We evaluated the impact on KPC-KpBSI mortality of the preemptive use of antibiotics active against KPC-*K.pneumoniae*, as opposed to inactive or standard empiric antibiotics, for the empiric treatment of febrile neutropenia episodes in patients with hematological malignancy identified as KPC-*K.pneumoniae* intestinal carriers.

**Methods:**

We compared the outcomes of KPC-KpBSIs occurring in high-risk hematological patients known to be colonized with KPC-*K.pneumoniae*, during two time periods:

March2012-December2013 (Period 1, initial approach to KPC-*K.pneumoniae* spread) and January2017-October2018 (Period 2, full application of the preemptive strategy). The relative importance of the various prognostic factors that could influence death rates were assessed by forward stepwise logistic regression models.

**Results:**

KPC-KpBSI-related mortality in hematological patients identified as KPC-*K.pneumoniae* carriers dropped from 50% in Period 1 to 6% in Period 2 (p < 0.01), from 58 to 9% in acute myeloid leukemia carriers(p < 0.01). KPC-KpBSIs developed in patients identified as KPC-*K.pneumoniae* carriers were initially treated with active therapy in 56% and 100% of cases in Period 1 and Period 2, respectively (p < 0.01), in particular with an active antibiotic combination in 39 and 94% of cases, respectively(p < 0.01). The 61% of KPC-KpBSI observed in Period 1 developed during inactive systemic antibiotic treatment (none in Period 2, p < 0.01), fatal in the 73% of cases. Overall, KPC-KpBSI-related mortality was 88% with no initial active treatment, 11.5% with at least one initial active antibiotic (p < 0.01), 9% with initial active combination. Only the initial active treatment resulted independently associated with survival.

**Conclusions:**

In high-risk hematological patients colonized by KPC-*K.pneumoniae*, the empiric treatment of febrile neutropenia active against KPC-*K.pneumonia*e reduced KPC-KpBSI-related mortality to 6% and prevented fatal KPC-KpBSI occurrence during inactive systemic antibiotic treatment.

## Background

The mortality rate associated with KPC-*K.pneumoniae* bloodstream infection (KPC-KpBSI) in neutropenic patients with hematological malignancies is reported up to 60% [1–10]. The initial active antibiotic treatment is crucial for KPC-KpBSI survival [11, 12], especially in high-risk vulnerable patients such as acute leukaemia patients undergoing intensive chemotherapy, and hematopoietic stem cell transplant (HSCT) recipients [7–10]. A delay in KPC-KpBSI active antibiotic treatment is common in these patients since in most cases the recommended empiric antibiotic regimens for febrile neutropenia contain antibiotics without in vitro activity against carbapenem-resistant Enterobacteriaceae (CRE) [13, 14]. Colonization is a recognized predictive factor for KPC-KpBSI occurrence [10, 15, 16), and the prevalence of colonized hematological patients hospitalized for chemotherapy treatments is increasing in Italy and many other countries with high KPC-*K.pneumoniae* nosocomial diffusion [17]. On March 2012, KPC-*K.pneumoniae* spread at the Hematology Department of Sapienza University of Rome (Italy): we reported the 71% of KPC-KpBSI mortality rate, and a high rate of KPC-K*.pneumoniae* rectal carriers who developed KPC-KpBSI and died [8]. Good-quality studies on CRE-BSI treatment in neutropenic patients were not available at that time, and recommendations were not sufficiently focused   [13, 18]. We implemented the prompt identification of patients hospitalized who were KPC-*K.pneumoniae* carriers and we progressively increased the preemptive use of antibiotics active against KPC-*K.pneumoniae* for the empiric treatment of febrile neutropenia episodes in KPC-*K.pneumoniae* carriers. Options for CRE treatment are limited [19], and data on hematological patients scarce. Several issues pertain the clinical use of colistin, dosing, pharmacokinetics in critically ill patients, nephrotoxicity and uncertainties about susceptibility testing [12], moreover the emergence of resistance decreases the activity of colistin and other available antibiotics [20]. Currently, evidences and recommendations support the use of active antibiotic combinations to treat serious CRE infections [12, 21–23]. New combinations with β-lactamase inhibitors, including carbapenem/β-lactamase inhibitor combinations, represent therapeutic alternatives [24–26] to limit colistin use and improve CRE treatment. Good response rates are reported with the use of the ceftazidime-avibactam for targeted or salvage treatment of KPC-KpBSI in immunocompetent patients [24, 25], while data in the specific setting of patients with hematological malignancies are limited [27].

We evaluated the impact of the preemptive strategy on KPC-KpBSI-related mortality in high-risk hematological KPC-*K.pneumoniae* carriers comparing the initial approach to KPC-*K.pneumoniae* spread and the routine application of the preemptive use of active therapy for the empiric treatment of febrile neutropenia.

## Patients and methods

We compared KPC-KpBSI developed in patients with hematological malignancies identified as KPC-*K.pneumoniae* carriers attending the Hematology Department of Sapienza University of Rome between March 2012- December 2013 (Period 1) and January 2017-October 2018 (Period 2).

### Setting

The Hematology Department consists of 5 wards and 52 beds. The majority of patients are hospitalized for intensive chemotherapy treatments for acute leukemia and/or HSCT.

### Screening for KPC-*K.pneumoniae* colonization

From March 2012, when KPC-*K.pneumoniae* spread at the Hematology Department, the screening for KPC*-K.pneumoniae* rectal colonization [15] was performed weekly in patients hospitalized in the ward where KPC-*K.pneumoniae* was first isolated. From September 2012, the screening was extended to all patients hospitalized in the Hematology Department, from October 2013, rectal swabs were collected also prior to admission. From January 2018, hospitalized acute leukaemia patients were screened twice weekly. KPC*-K.pneumoniae* colonized patients underwent contact precautions.

### Empiric antibiotic treatments started within 4 h from febrile neutropenia onset

#### Standard treatment

Piperacillin-tazobactam (4.5 g q8h) with or without tigecyclin (100 mg loading dose, then 50 mg q12h) combination  [14].

#### Empiric treatments used for KPC-*K.pneumoniae* carriers

Combination of colistin (9 million international units [IU] loading dose, then 4.5 million UI q12h) with tigecycline (100 mg loading dose, then 50 mg q12h) and/or gentamycin (3 mg/kg/die once-a-day), high-dose carbapenems (meropenem 2 g q8h), double carbapenem (meropenem plus ertapenem 1 g once-a-day) and, from September 2017, ceftazidime-avibactam (2.5 g q8h) as monotherapy or combined with tigecycline and/or gentamycin.

### Clinical data recorded

Data collected from Period 1 and Period 2: (a) number of KPC-KpBSI occurred in KPC*-K.pneumoniae* carriers. (b) number of hospitalized patients identified as KPC*-K.pneumoniae* carriers. (c) type of hematological malignancy and chemotherapy treatment administered in the colonized patients with KPC-KpBSI. (d) length of neutropenia (< 1000 neutrophils/cmm) and profound neutropenia (< 100 neutrophils/cmm) episodes. (e) clinical presentation of KPC-KpBSI. f) antibiotic treatment. g) KPC-KpBSI outcome.

### Microbiological studies

In Period 1, species identification and MIC determination of *K.pneumoniae* isolates were performed using the BD Phoenix automated microbiology system (Becton Dickinson Italia S.p.A Milano, Italy). MICs of imipenem, meropenem, ertapenem, gentamicin, colistin and tigecycline were also evaluated using Etest (BioMerieux Italia S.p.A., Firenze, Italy). In Period 2, species identification was performed using MALDI-TOF and susceptibility testing was determined using the automated VITEK2 system (bioMérieux, Marcy-l’Étoile, France). *K.pneumonia* blood isolates KPC genetic mechanism was determined with in vitro real-time PCR assay Xpert Carba-R assay (Cepheid, Sunnyvale,CA). For the 34 KPC-*K.pneumonia* blood isolates, MICs for imipenem, meropenem, ertapenem colistin, tigecycline, gentamicin and ceftazidime-avibactam were determined by broth microdilution (Sensititre Gram Negative MIC Plate, ThermoFischer Scientific, USA) and results were interpreted in accordance with the 2019 breakpoints proposed by the European Committee on Antimicrobial Susceptibility Testing(EUCAST 2019) [28].

### Statistical analysis

Continuous variables were compared using the Kruskal–Wallis test. Categorical variables were compared with the Chi-Square test corrected for continuity or the Fisher’s exact test when indicated; 95% confidence intervals (CIs) for the differences of means and proportions were calculated. Forward stepwise logistic regression models were used to assess the relative importance of the various prognostic factors that could influence the occurrence of death in patient populations [7, 8]: acute myeloid leukemia (AML), intensive chemotherapy, shock at onset, breakthrough KPC-KpBSI, active initial therapy. The statistical calculations were carried out using the SPSS statistical package (SPSS for Windows, Release 15.0).

## Results

Eighteen KPC-KpBSI developed among 27 patients identified as KPC*-K.pneumoniae* carriers in Period 1 and 16 KPC-KpBSI among 88 KPC*-K.pneumoniae* carriers in Period 2 were compared. The rate of KPC*-K.pneumoniae* colonized patients who developed KPC-KpBSI reduced from 67% (18/27) in Period 1 to 11% (16/88) in Period 2, p < 0.01 (Table 1).

**Table 1:** Characteristics of patients with hematological malignancies KPC-*K.pneumoniae* carriers who developed KPC-*K.pneumoniae* BSI

	Period 1: March 2012–Dec 2013 N 18	Period 2: Jan 2017–Oct 2018 N 16	p-value (absolute difference; 95% CI)
Male	7 (39%)	6 (37%)	0.2 (0.02; -0.31 to 0.34)
Mean age, years (range)	47.3 (28–68)	50.8 (3–68)	0.07 (-3.5; -13.2 to 6.24)^a^
Acute leukemia	16 (89%)	12 (75%)	0.2 (0.14; -0.11 to 0.39)
- Myeloid	12/16 (75%)	10/12 (83%)	0.4 (0.08; -0.38 to 0.21)
Other hematological diseases	2 (11%)	4 (25%)	0.2 (0.14; -0.39 to 0.11)
Intensive chemotherapy	15 (83%)	12 (75%)	0.4 (0.08; -0.19 to 0.35)
Allogeneic HSCT	1 (6%)	4 (25%)	0.1 ( -0.19; -0.43 to 0.04)
Other treatment	2 (11%)	0 (0%)	0.2 (0.11; -0.03 to 0.25)
Length of neutropenia episode (mean days, range)			
- With < 1000 neutrophils/cmm	17.6 (5–35)	21.1 (11–40)	0.3 (-3.50; -9.2 to 2.2)^a^
- With < 100 neutrophils/cmm	8.4 (0–17)	12.5 (6–25)	0.07 (-4.1; -7.86 to -0.31)^a^
N. of KPC-*K.pneumonia* carriers identified during the period	27	88	
Carriers who developed *K.pneumonia* BSI	18 (67%)	16 (11%)	< 0.01 (0.48; 0.29 to 0.68)

^a^Continuous variables were compared using Kruskal–Wallis test

As shown in Table 1, the characteristics of the KPC*-K.pneumonia*e carriers with KPC-KpBSI were similar in the two periods: the large majority of patients had acute leukaemia, mostly acute myeloid leukaemia (AML) and underwent intensive chemotherapy, the length of neutropenia and profound neutropenia episodes were comparable. In both periods, KPC-KpBSI occurred during profound neutropenia in the majority of cases, and presented with shock in about half of the patients (Table 2). The 61% (11/18) of KPC-KpBSI occurred in Period 1 compared with none in Period 2, developed in patients who were already receiving inactive systemic antibiotics (from 4.2 mean days, range 2–8) started for the empiric treatment of febrile neutropenia (p < 0.01).

**Table 2:** Clinical characteristic of KPC-*K.pneumoniae* BSI

	Period 1: March 2012–Dec 2013 N 18	Period 2: Jan 2017–Oct 2018 N 16	p-value (absolute difference; 95% CI)
KPC-*K.pneumoniae* BSI onset			
-Shock	10 (56%)	7 (44%)	0.73 (0.12; -0.21 to 0.45)
-Neutropenia			
< 1000 neutrophils/cmm	18 (100%)	16 (100%)	
< 100 neutrophils/cmm	14 (78%)	15 (94%)	0.40 (-0.16; -0.38 to 0.06)
-BSI developing under inactive antibiotic treatment	11 (61%)	0	< 0.01 (0.61; 0.38 to 0.83)
Initial active treatment	10 (56%)	16 (100%)	** < **0.01 (-0.44; -0.67 to -0.21)
-Combination	7 (39%)	15 (94%)	< 0.01 (-0.54; -0.80 to -0.29)
with colistin	7 (39%)	4 (25%)	0.31 (0.13; -0.17 to 0.44)
with ceftazidime-avibactam	0	11 (69%)	< 0.01 (-0.68; -0.91 to -0.46)
-Monotherapy	3 (17%)	1 (6%)	0.34 (0.11; -0.10 to 0.31)
Tigecyclin^a^	3 (17%)	0	0.13 (0.17; -0.005 to 0.33)
Ceftazidime/avibactam	0	1 (6%)	0.47;(-0.06; -0.18 to 0.05)
Fatal KPC-*K.pneumoniae* BSI	9 (50%)	1(6%)	< 0.01 (0.44; 0.17 to 0.69)
-Death within 96 h	4 (22%)	0	0.06 (0.22; 0.03 to 0.41)
-Shock	7 (39%)	0	< 0.01 (0.39; 0.16 to 0.61)
-BSI developing during inactive antibiotic treatment	8 (44%)	0	< 0.01 (0.44; 0.21 to 0.67)
-Inactive initial treatment	7 (39%)	0	< 0.01 (0.39; 0.16 to 0.61)
-Acute myeloid leukemia	7 (39%)	1 (6%)	< 0.01 (0.33; 0.07 to 0.58)

^a^Combined with piperacillin/tazobactam as empiric treatment of febrile neutropenia [14]

As shown in Table 2, the 56% of KPC-KpBSI occurred in Period 1 and 100% of those occurred in Period 2 in KPC*-K.pneumoniae* carriers were treated with active therapy from the very onset, at least one active antibiotic, preemptively administered as empiric treatment of febrile neutropenia (p < 0.01). An active combination was used in 39% and 94% of cases in Period 1 and Period 2, respectively (p < 0.01) (Table 2). In particular, 7 of 18 (39%) KPC-KpBSI patients in Period 1, and 4 of 16 (25%) patients in Period 2, previously screened negative during the same hospitalization, received active antibiotics as empiric treatment because they had the first microbiological result showing KPC*-K.pneumoniae* colonization available on the same day of febrile neutropenia onset.

KPC-KpBSI-related mortality reduced from 50% in Period 1 (9/18) to 6% in Period 2 (1/16), p < 0.01. Notably, KPC-KpBSI mortality rate in AML carriers decreased from 44% (7/16) in Period 1 to 8% (1/12) in Period 2, p < 0.01 (Table 1). The rate of patients who died for KPC-KpBSI among those identified as KPC*-K.pneumoniae* carriers, reduced from 33% (9/27) in Period 1 to 1.1% (1/88) in Period 2, p < 0.01.

In Period 1, fatal KPC-KpBSI presented with shock in 7 (78%) cases and death occurred within 1 week in 6 patients (66%), within 96 h in 4 of them. Fatal KPC-KpBSI developed during inactive systemic antibiotics in 8 cases (89%)—the overall mortality rate of KPC-KpBSI developed during inactive antibiotics was 73%- and fatal KPC-KpBSI were initially treated with inactive antibiotics in 7 cases (78%). In Period 2, one AML patient with severe allogeneic-HSCT-related complications, died 10 days after KPC-KpBSI onset.

Overall, KPC-KpBSI observed in Period 1 and Period 2 were fatal in 29% (10/34) of cases: KPC-KpBSI mortality rate was 88% in patients not receiving initial active antibiotics, 11.5% in patients initially treated with at least one active antibiotic (p < 0.01). Notably, KPC-KpBSI mortality rate was 25% in patients treated with a single active antibiotic and 9% in those treated with active combinations. At univariate analysis, initial inactive treatment, KPC-KpBSI developing during inactive antibiotics and KPC-KpBSI occurrence in Period 1, resulted risk factors for mortality (Table 3). In the forward stepwise logistic regression analysis (Table 4), if the initial active therapy was not considered (Model 1), the mortality rate of KPC-KpBSI developing during inactive antibiotics resulted independently associated with death, but when the initial active therapy was added (Model 2), it resulted the only factor independently associated with survival. In both models, AML as underlying diseases, shock and intensive chemotherapy were not predictors of mortality.

**Table 3:** Univariate Analysis of Risk Factors for Mortality

	Survivors: N 24	Non-survivors: N 10	p-value (absolute difference; 95% CI)
Male	8 (33%)	5 (50%)	0.4 (-0.17; -0.52 to 0.19)
Mean age, years (range)	47.7(3–68)	52 (35–68)	0.6 (-4.30; -14.9 to 6.32)^a^
Acute leukemia	19 (79%)	9 (90%)	0.41 (-0.11; -0.35 to 6.32)
- Myeloid	14 (58%)	8 (80%)	0.21 (-0.22; -0.53 to 0.10)
Other hematological disease	5 (21%)	1 (10%)	0.41 (-0.21; -0.53 to 0.10)
Intensive chemotherapy	20 (83%)	8 (80%)	0.58 (0.03; -0.25 to 0.32)
Other chemotherapy	1 (4%)	–	0.70 (0.04;-0.03 to 0.12)
Allogeneic stem cell transplant	3 (12%)	2 (20%)	0.46 (-0.08; -0.35 to 0.20)
Length of neutropenia episode (mean days, range)			
- With < 1000 neutrophils/mmc	19.4 (5–40)	18.9 (8–35)	0.70 (0.50; -5.90 to 6.90)^a^
- With < 100 neutrophils/mmc	11.2 (5–25)	8.3 (0–17)	0.29 (2.90; -3.29 to 9.09)^a^
Onset of KPC-*K.pneumoniae* BSI			
- Shock	10 (42%)	7 (70%)	0.12 (-0.28;-0.62 to 0.06)
-< 100 neutrophils/mmc	21 (87%)	8 (80%)	0.61 (0.07; -0.20 to 0.35)
- KPC*-K.pneumoniae* BSI developing during inactive antibiotic treatment^b^	3 (12.5%)	8 (80%)	< 0.01 (-0.67;-0.95 to -0.39)
Active initial treatment	23 (96%)	3 (30%)	< 0.01 (-0.76; -1.02 to -0.50)
- Combination	20 (83%)	2 (20%)	< 0.01 (0.63; 0.34 to 0.92)
With colistin	10	1	0.07 (0.32; 0.04 to 0.58)
With ceftazidime/avibactam	10	1	0.07 (0.32; 0.04 to 0.58)
- Monotherapy	3 (12.5%)	1 (10%)	0.66 (0.02; -0.20 to 0.25)
Tigecycline^c^	2 (8.5%)	1 (10%)	0.66 (-0.16;-0.23 to 0.20)
Ceftazidime/avibactam	1 (4.5%)	–	0.70 (0.4; -0.03 to 0.12)
Study period			
- Period 1 (March 2012–Dec 2013)	9 (37.5%)	9 (90%)	< 0.01 (− 0.52; − 0.79 to − 0.25)
- Period 2 (Jan 2017–Oct 2018)	15 (62.5%)	1 (10%)	< 0.01 (0.52; 0.25 to 0.79)

^a^Continuous variables were compared using Kruskal–Wallis test

^b^Developed in KPC-*K.pneumoniae* carriers already receiving standard empiric antibiotic treatment

^c^Combined with piperacillin/tazobactam as empiric treatment of febrile neutropenia [14]

**Table 4:** Multivariate Models of risk factors for 30 days crude mortality in patients population (Forward Stepwise logistic regression)

	OR (CI 95%)	p-value
MODEL 1		
KPC*-K.pneumoniae* BSI developing during inactive antibiotic treatment^a^	28 (3.9 to 199)	0.001
Acute myeloid leukemia	Not included in the Model	
Shock at onset	Not included in the Model	
Intensive chemotherapy	Not included in the model	
MODEL 2		
Initial active treatment	0.019 (0.002 to 0.20)	0.001
KPC*-K.pneumoniae* BSI developing during inactive antibiotic treatment^a^	Not included in the model	
Acute myeloid leukemia	Not included in the model	
Shock at onset	Not included in the model	
Intensive chemotherapy	Not included in the model	

^a^Developing in KPC-*K.pneumoniae* carriers receiving standard empiric antibiotic treatment

The susceptibilities to antibiotics of the 34 KPC-*K.pneumoniae* blood isolates are shown in Table 5. According to 2019 EUCAST breakpoints (38), colistin resistant strains were 33 and 22.5% in Period 1 and Period 2, respectively. A higher tigecyclin activity was observed in Period 2, all the isolates in Period 1 compared to 50% in Period 2 had tigecyclin MIC > 0.5 mg/L (p < 0.01). All isolates resulted susceptible to ceftazidime-avibactam.

**Table 5:** Susceptibilty of *KPC-K.pneumoniae* blood isolates determined by broth microdilution according to 2019 EUCAST breakpoints (38)

	Period 1		Period 2	
N of blood isolates	18		16	
	MIC range (mg/L)	N(%) of susceptible isolates	MIC range (mg/L)	N (%) of susceptible isolates
Colistin	0.25–32	11 (67)	0.5–4	14 (87.5)
Tigecycline	2–8	0	0.5–2	8 (50)
Gentamicin	1.5– >256	8 (44)	1– >8	8 (50)
Ceftazidime-avibactam	0.5/4–8/4	18 (100)	0.5/4–8/4	16 (100)
Meropenem	> 32	0	> 32	0
Imipenem	> 32	0	> 32	0
Ertapenem	> 32	0	> 32	0

## Discussion

Rectal colonization is a recognized predictive factor for CR-KpBSI occurrence[15, 16]. Patients with hematological malignancies colonized with CR-*K.pneumoniae*, mostly AML patients, are at high risk of developing BSI [4, 8, 10]. In this population, the reported CR-KpBSI mortality rate is dramatic [1–10], mainly in acute leukaemia patients [7, 8]. AML, intensive chemotherapy and severe and/or prolonged neutropenia were associated with CR-KpBSI-related death [7, 8], and septic shock and acute respiratory failure resulted predictors of mortality [7].

This study focuses on KPC-KpBSI-related mortality in high-risk patients with hematological malignancies known to be colonized by KPC-*K.pneumoniae.* The prompt identification of KPC-*K.pneumoniae* carriers, and the preemptive antibiotic strategy applied to all the carriers -KPC-*K.pneumoniae* active therapy for the empiric treatment of febrile neutropenia- was associated with the drop of KPC-KpBSI-related mortality from 50%, observed between March 2012-December 2013, to 6% recorded 3 years later, between January 2017-October 2018. To our knowledge, this is the lowest rate reported so far.

KPC*-K.pneumoniae* colonized patients who developed BSI in the two periods had comparable high risk factors for KPC-KpBSI-related death: the majority had acute leukaemia, mainly AML, and had received intensive chemotherapy developing profound and prolonged neutropenia, KPC-KpBSI presented with shock in a similar proportion of patients, but KPC-KpBSI mortality rate was significantly higher in Period 1. Initial treatment with active antibiotics is crucial for survival to CR-KpBSI [7, 8, 11, 12], and the 78% of patients who died from KPC-KpBSI in Period 1 had received inactive initial antibiotic treatment.

KPC-KpBSI treatment based on culture results presents a high risk of delaying or never receiving appropriate therapy [1, 5]. In hematological neutropenic KPC*-Kpneumoniae* carriers at high risk of KPC-KpBSI [8–10], the initial appropriate treatment can be ensured by the preemptive administration of active therapy at any febrile episode. This preemptive approach was fully applied in Period 2, all the KPC-KpBSI patients received initial active therapy and KPC-KpBSI-related mortality dropped to 6%, as already observed applying the same strategy on a small population of HSCT carriers [9]. The multivariate analysis confirmed that only the initial active antibiotic treatment was independently associated with survival and, differently from other reports [7, 8], AML and shock were not predictors of mortality.

The role of initial active treatment in hematological patients with CR-KpBSI is critical. We previously reported the 90% of mortality in patients not receiving initial active treatment [8]; another study observed inappropriate initial treatment in the 78% of non-survivors, associated with 21-day mortality and significant predictor of mortality [7]. In the present study the overall KPC-KpBSI mortality rate in KPC*-Kpneumoniae* carriers not receiving initial active treatment was 88%, but reduced to 13% in those initially treated with at least one active antibiotic, and even to 9% with initial active combinations. The combination of active drugs had already been associated with a lower mortality rate [5] and independently associated with survival [7], and as a matter of fact in Period 2 all but one KPC-KpBSI patients received an active combination as initial treatment.

We reported an increased number of carriers identified during Period 2. In our opinion, this data is mainly related to the increase in nosocomial transmission of KPC-*K.pneumoniae* in Italy where, despite the application of control measures, the prevalence of hospitalized patients colonized by KPC-*K.pneumoniae* has progressively grown over the recent years. The progressive implementation of the screening program in our Department could have further favored the identification of all colonized patients.

We reported a reduced rate of KPC-KpBSI among KPC-*K.pneumoniae* carriers in Period 2. This data may be explained with the preemptive administration of active antibiotics at the onset of any febrile neutropenia episode in carriers. This strategy, fully applied in Period 2, avoiding the intestinal overgrowth of the colonizing KPC-*K.pneumoniae* during inactive antibiotics and their subsequent blood-stream diffusion through the damaged mucosa, could have prevented KPC-KpBSI occurrence. During Period 2, no KPC-KpBSI occurred during inactive empiric antibiotic treatment as was observed in 61% of those detected in Period 1, moreover fatal in the 72% cases. The prevention of KPC-KpBSI onset during inactive treatment burdened by high mortality [8], may have strongly contributed to the overall reduction in KPC-KpBSI-related mortality reported in Period 2.

In high prevalence areas, active surveillance of the gastrointestinal tract is critical to avoid KPC*-K.pneumoniae* nosocomial diffusion [8–10, 16], particularly in hematological wards where several conditions favor KPC*-K.pneumoniae* spread [8]. In our experience we focused on the search and prompt detection of KPC-*K.pneumoniae* carriers, with the purpose of identifying the patients at the highest risk to develop KPC-KpBSI who would mostly benefit from the preemptive active strategy, obtaining a targeted initial treatment for KPC-KpBSI. In patients with hematological malignancies we strongly recommend the careful screening for KPC*-K.pneumoniae* rectal colonization, which represented the cornerstone of the preemptive strategy we customarily applied in Period 2: all KPC-KpBSI analyzed in the two study Periods developed in patients known as KPC*-K.pneumoniae* carriers but, in Period 2 all KPC-KpBSI were initially treated with active therapy. It is to be noted that in both periods the high frequency of rectal screening avoided the delay in the active initial treatment in unrecognized new carriers from a third to a quarter of KPC-KpBSI cases.

Finally, the reduction in the probability of death from KPC-KpBSI led us to be less concerned about the risk of fatal KPC-KpBSI in appropriately scheduling or prosecuting intensive chemotherapeutic and/or HSCT programs in hematological patients identified as KPC-*K.pneumoniae* carriers.

Some considerations are needed. The preemptive strategy certainly increases the use of active drugs, and consequently a de-escalation of empirical active treatments should be carried out if KPC-*K.pneumonia* infection is not confirmed. Nephrotoxicity [19], especially in hematological patients receiving many other toxic drugs -chemotherapeutics, immunosuppressants, antifungals- and the emergence of resistance [17, 19, 20] observed in 26% of our KPC-*K.pneumonia* blood isolates, makes colistin role uncertain. The availability of Ceftazidime-avibactam, widely used against KPC-*K.pneumonia* infections with lower toxicity than colistin or aminoglycosides [19–25, 27] favored the full application of the preemptive strategy in Period 2; Ceftazidime-avibactam proved active against all our KPC*-K.pneumoniae* blood isolates but resistance reported during [29] and independently [30] from previous ceftazidime-avibactam exposure, is troubling. New combinations with β-lactamase inhibitors, including carbapenem/β-lactamase inhibitor combinations [26], could represent new therapeutic alternatives also in the setting of hematological patients.

The monocentric design, the low number of patients included and the availability of Ceftazidime-avibactam only after 2017, represent a limitation to the present report, and prospective larger studies are needed to confirm and generalize the results. Good-quality prospective randomized studies on CRE infections management are still lacking, also in neutropenic haematological patients, and therefore our strategy -search of carriers and preemptive active treatment- obtained a noteworthy and promising reduction of KPC-KpBSI-related mortality in this population.

## Conclusion

In high-risk haematological patients colonized with KPC-*K.pneumoniae,* the preemptive use of antibiotics active against KPC-*K.pneumoniae,* in particular active combinations, as empiric treatment of febrile neutropenia resulted in a drop to 6% of KPC-KpBSI mortality rate, reduction of KPC-KpBSI occurring during inactive antibiotics and probability of death for KPC-KpBSI, including AML patients.

## Data Availability

Clinical data can be found in the medical records of patients stored at the institutional repository of the Hematology Unit, Department of Translational and Precision Medicine, Sapienza University of Rome, Italy. Microbiological data can be found in the electronic register of the internal Laboratory of Microbiology of the Hematology Unit, Department of Translational and Precision Medicine, Sapienza University of Rome, Italy. Each patient included in the study was given a code for the subsequent analysis. The datasets used and/or analysed during the current study are available from the corresponding author on reasonable request.

## Abbreviations

KPC-KpBSI: KPC-*K.pneumoniae* bloodstream infection
CRE: Carbapenem-resistant Enterobacteriaceae
HSCT: Hematopoietic stem cell transplant
AML: Acute myeloid leukaemia
